# The Influence of a Coherent Annotation and Synthetic Addition of Lung Nodules for Lung Segmentation in CT Scans

**DOI:** 10.3390/s22093443

**Published:** 2022-04-30

**Authors:** Joana Sousa, Tania Pereira, Inês Neves, Francisco Silva, Hélder P. Oliveira

**Affiliations:** 1 INESC TEC—Institute for Systems and Computer Engineering, Technology and Science, 4200-465 Porto, Portugal; tania.pereira@inesctec.pt (T.P.); francisco.c.silva@inesctec.pt (F.S.); helder.f.oliveira@inesctec.pt (H.P.O.); 2 FEUP—Faculty of Engineering, University of Porto, 4200-465 Porto, Portugal; 3 ICBAS—Abel Salazar Biomedical Sciences Institute, University of Porto, 4050-313 Porto, Portugal; up201704832@edu.icbas.up.pt; 4 FCUP—Faculty of Science, University of Porto, 4169-007 Porto, Portugal

**Keywords:** deep learning, data augmentation, annotation homogeneity, lung segmentation, lung diseases

## Abstract

Lung cancer is a highly prevalent pathology and a leading cause of cancer-related deaths. Most patients are diagnosed when the disease has manifested itself, which usually is a sign of lung cancer in an advanced stage and, as a consequence, the 5-year survival rates are low. To increase the chances of survival, improving the cancer early detection capacity is crucial, for which computed tomography (CT) scans represent a key role. The manual evaluation of the CTs is a time-consuming task and computer-aided diagnosis (CAD) systems can help relieve that burden. The segmentation of the lung is one of the first steps in these systems, yet it is very challenging given the heterogeneity of lung diseases usually present and associated with cancer development. In our previous work, a segmentation model based on a ResNet34 and U-Net combination was developed on a cross-cohort dataset that yielded good segmentation masks for multiple pathological conditions but misclassified some of the lung nodules. The multiple datasets used for the model development were originated from different annotation protocols, which generated inconsistencies for the learning process, and the annotations are usually not adequate for lung cancer studies since they did not comprise lung nodules. In addition, the initial datasets used for training presented a reduced number of nodules, which was showed not to be enough to allow the segmentation model to learn to include them as a lung part. In this work, an objective protocol for the lung mask’s segmentation was defined and the previous annotations were carefully reviewed and corrected to create consistent and adequate ground-truth masks for the development of the segmentation model. Data augmentation with domain knowledge was used to create lung nodules in the cases used to train the model. The model developed achieved a Dice similarity coefficient (DSC) above 0.9350 for all test datasets and it showed an ability to cope, not only with a variety of lung patterns, but also with the presence of lung nodules as well. This study shows the importance of using consistent annotations for the supervised learning process, which is a very time-consuming task, but that has great importance to healthcare applications. Due to the lack of massive datasets in the medical field, which consequently brings a lack of wide representativity, data augmentation with domain knowledge could represent a promising help to overcome this limitation for learning models development.

## 1. Introduction

Lung cancer, associated with high rates of incidence and mortality, has registered 2.2 million new cases and 1.79 million deaths in 2020, and it is one of the leading causes of cancer-related deaths [1]. Several factors can contribute to the development of this pathology, such as age, dietary habits, air pollution, and genetic predispositions, yet what contributes the most is tobacco intake [2]. Lung cancer can be classified into two major hystological types: non-small-cell lung cancer (NSCLC), which represents the vast majority of cases, about 85%, and small-cell lung cancer (SCLC). A large number of patients are diagnosed when symptoms arise, which is usually associated to an advanced stage of the condition, resulting in lower 5-year survival rates, primarily caused by the late detection of the disease. For this reason, screening has an important and key role as it allows for a prompt detection and thus possibly preventing the further dissemination of the disease. Computed tomography (CT) is one of the available imaging tools that is usually used in screening, diagnosis, and treatment planning, which for that reason plays a significant function in the evaluation of lung cancer progression [3].

The evaluation of CT images for the detection and assessment of lung cancer constitutes a time-demanding task and is highly dependent on the physician’s interpretation. In that sense, AI-based systems, namely computer-aided diagnosis (CAD) methodologies, have a significant impact, since they can give a much faster and automatic response that also serves as a complementary assistance to the medical experts. In some CAD systems designed for lung cancer, the segmentation of the lung represents the first stage in order to discard any irrelevant information and to enable the systems to focus solely on the parenchyma of the lungs [4]. Due to its susceptibility, the respiratory system is prone to several pathologies, each one with its own pathophysiological characteristics, which results in a wide spectrum of lung parenchyma heterogeneites. Therefore, the segmentation of the lung is a very complicated task and, since it precedes phases, such as the classification of a disease, it is extremely important that the segmentation is correctly performed so it does not lead to an inaccurate diagnosis.

Among the several approaches proposed for the segmentation of the lung, deep learning (DL)-based methods have shown the capability of delivering good results in comparison to previous and traditional methodologies. Khanna et al. [5] developed a Residual U-Net for the lung segmentation in CT images, which is the result of ResNet [6] and U-Net [7] architectures. Data augmentation (DA) was performed to improve the number of training images, and a connected component algorithm was implemented to remove non-lung regions as a final step. The loss function used was based on the Dice similarity coefficient (DSC), and for training the LUng Nodule Analysis 2016 (LUNA16) and the VESsel SEgmentation in the Lung 2012 (VESSEL12) datasets were used, whereas the HUG-ILD dataset was used in the test. An average DSC of 0.9868 was achieved using the ResNet50 architecture. Tan et al. [8] presented a generative adversarial network for the segmentation of the lung, denominated LGAN, which includes a generator network that yields binary lung masks, and a discriminator network, which discriminates between the ground-truth and the produced lung segmentation. For the discriminator, five different designs were developed and the LGAN with the regression network was the one that demonstrated a better performance. The Lung Image Database Consortium-Image Database Resource Initiative (LIDC-IDRI) and the QIN Lung CT datasets were used and both were divided into training and test subsets. The developed model achieves an intersection over union (IoU) of 0.9230 and 0.9380 on the former and the latter subsets, respectively.

In our previous work [9], a network resulting from the combination of U-Net and ResNet34 architectures was used and developed in a cross-cohort dataset, having achieved DSC values above 0.9300 for all four test databases. The model was able to produce good segmentation masks overall and it showed the capability to segment intricate patterns of interstitial lung diseases; nonetheless, it failed to recognize a great proportion of lung masses as part of the lung. Moreover, the presence of errors and discrepancies in the lung annotations, namely the exclusion of lung nodules between datasets led to inaccurate results and contributed to a lack of ability to correctly identify these structures. With that in mind, there was a need to create coherent training data that would allow the development of models that would output consistent segmentations. In addition, as mentioned above, given that the main challenge of the previous model was the segmentation of the lung masses, a question arose as to whether a data augmentation approach based on the synthetic addition of lung nodules would enhance a model’s ability to carry out that task. The performance achieved by deep learning models depends not only on the complexity of the network and the size of their training sets, but also on the heterogeneity covered in the training sets and the quality of their labels. In the medical field, obtaining large and representative datasets with accurate labeling is challenging, and far often label noise is present. Scarce annotations and weak annotations, a category that includes noisy annotations and image-level annotations, are some of the limitations found within segmentation datasets. In the particular case of label noise, this type is related to imperfections in the delineations of the segmentation masks, and it may be caused by the errors of the annotators or by discrepancies between the annotators that follow different guidelines, leading to inter-reader variability. If not addressed, this issue can lead to a degradation in the performance of the learning models [10,11]. In addition, given the heterogeneity of medical imaging patterns and the lack of diversity in the training data, the capability of the models to generalize is negatively affected [3,12]. In this work, two main contributions were implemented to improve the previous segmentation model: the label noise influence was decreased due to the uniformization of the lung masks, and the addition of the data augmentation creates a combination of pathologies usually found in lung cancer patients, generating more variability in the training and a better generalization of the segmentation model.

The structure of the paper is as follows: the Materials and Methods section describes the multiple datasets used in this study and their original annotation protocols. An objective data correction protocol was presented to create coherent segmentations of the lung structures along with the different datasets. A data augmentation process with domain knowledge was proposed. The integration of the described steps generates four training sets, described in the Experiment Design subsection. The Results section shows the performance of the segmentation models by three metrics and the Discussion section performs a comparison and impact assessment of the experiment design. The section Limitations describes the limitations found in this work.

## 2. Materials and Methods

**Figure 1:**
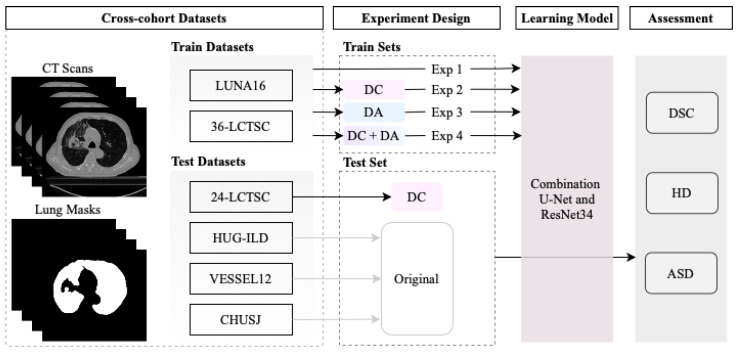
Overview of the segmentation model development using four experiments: trained with original annotations of the datasets in Experiment 1 (Exp 1); the corrected annotations in Experiment 2 (Exp 2); with data augmentation in Experiment 3 (Exp 3); and combining the corrected annotations with data augmentation in Experiment 4 (Exp 4). Three objective metrics were implemented to evaluate the performance of the segmentation model: the similarity coefficient (DSC), Hausdorff distance (HD), and average symmetric surface distance (ASSD).

This section presents the multiple datasets used in the current study, the data annotation correction, data augmentation, and performance evaluation of the lung segmentation model. The pipeline of the work developed is represented in Figure 1.

### 2.1. Datasets

Due to the importance of the capability of a model to be robust to the multiple heterogeneities of the pulmonary diseases, five datasets were collected, four public (Lung CT Segmentation Challenge (LCTSC) 2017 [13], LUng Nodule Analysis 2016 (LUNA16) [14], University Hospitals of Geneva-Interstitial Lung Disease (HUG-ILD) [15], and VESsel SEgmentation in the Lung 2012 (VESSEL12) [16]) and one private (Centro Hospitalar e Universitário de São João (CHUSJ)), comprising a wide variety of lung patterns. The main characteristics of the datasets used in this work are represented in Table 1.

### 2.2. Mask Segmentation

#### 2.2.1. Original Masks

Table 2 describes, for each dataset, the pathologies comprised in their data and the original protocols used for the segmentation masks.

#### 2.2.2. Mask Correction Protocol

Due to the different origins and purposes of the annotation protocols from the datasets, there was a need to create uniform annotations of the lung regions. For this objective, all the original segmentations were reviewed following specific rules that make the masks used for training and evaluation coherent and appropriate for future automatic applications on lungs with possible multiple diseases. The review of the annotations was made under supervision of a medical student.

The selected area was obliged to some rules based on fundamental anatomic and radiology features, such as:

- The lung is surround by the bones (clavicle, sternum, ribs, and vertebra) and muscles of the chest wall, the pleura, and the mediastinum (trachea, esophagus, pericardium, heart, and main vessels);
- On CT scans, airways are characterized as low density, whereas all the other structures are observed in variable higher densities;
- The apex and base of the lung are harder to define due to the overlapping of other structures, such as the abdominal organs. Key factors to differentiate were the pleura and the low density of the lung in contrast to the subcutaneous tissue near the lung apex and the abdominal organs distal to the lung base.

The main corrections were:

- Excluding the upper airways, such as the trachea;
- Excluding the main bronchi;
- Defining the hilum, which contains structures such as the bronchi and pulmonary and systemic vessels. It is contiguous to the mediastinum and, for that reason, it can be harder to define. The main characteristic was to acknowledge the hilum as the root of the lung and, for that reason, the hilum structures were surrounded by lung tissue;
- Including the lung nodules, which, in the majority of cases, appear as a well defined higher density area. Peripheral lung nodules constitute a harder group of nodules to define and, in this case, the key factors were to evaluate the different densities between the chest wall and the nodule, as well to find the fine well-defined line that characterizes the pleura.

**Figure 2:**
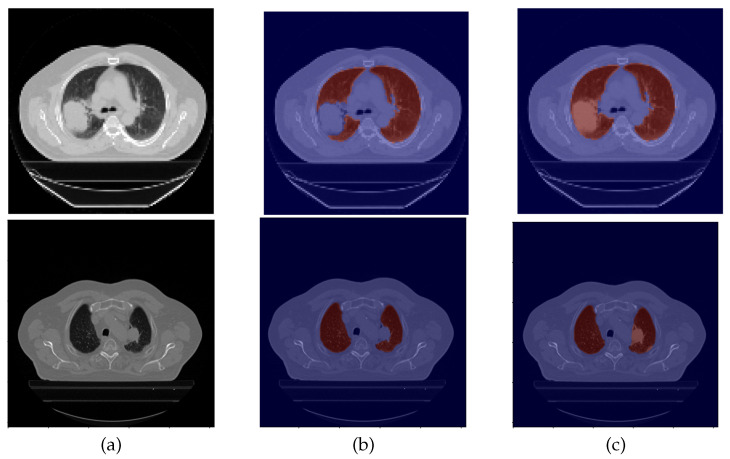
Example of a data correction. From left to right: (**a**) CT original image; (**b**) the original lung mask, which excludes the nodule; (**c**) and the corrected lung mask, which includes the nodule.

An example of data correction (DC) is displayed in Figure 2.

### 2.3. Pre-Processing

In the pre-processing phase, first, the images were submitted to a min-max normalization and then they were resized to a smaller dimension. Regarding the normalization step, the pixels of the 2D CT slices, expressed in Hounsfield units (HU), represent the X-ray attenuation of a certain body structure and the values of these pixels can lie in the range from −1000 to 1000 HU, approximately [17]. Therefore, the images underwent a min-max normalization to convert that range into 0, 1, using −1000 and 400 HU as the lower and upper limits, respectively. With respect to the second step, the images’ original sizes were rescaled to 128 × 128 via bilinear interpolation to reduce the computational burden. It is important to denote that the images’ dimensions were not altered in the z-direction.

### 2.4. Segmentation Model

By making use of the network developed in our previous work [9], a hybrid structure consisting of the combination of U-Net and ResNet34 networks was used, since it demonstrated the capability to produce good segmentation masks of the lungs; therefore, no additional changes were made to its architecture for this work. The encoder path of the structure is composed of residual blocks that follow the ResNet34 configuration, comprising convolutions, batch normalization, parametric ReLU activation (PReLU), and short connections in each residual unit. On the other hand, the decoder path includes U-Net blocks of 2D upsampling via 2D transpose convolutions, concatenation, convolutions, and ReLU activation. Lastly, the output of the last block is submitted to a 1 × 1 convolution with a sigmoid activation that produces the probability map for the segmentation of the lung.

### 2.5. Data Augmentation

**Figure 3:**
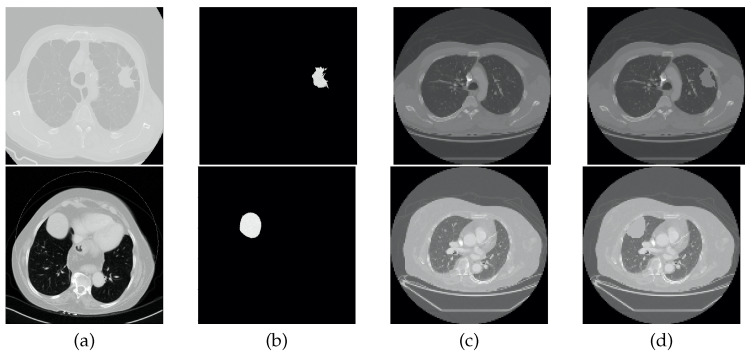
Example of data augmentation. From left to right: (**a**) original CT image containing a nodule; (**b**) ROI of the nodule; (**c**) original CT image without nodules; (**d**) and synthetic CT image that includes a nodule.

**Figure 4:**
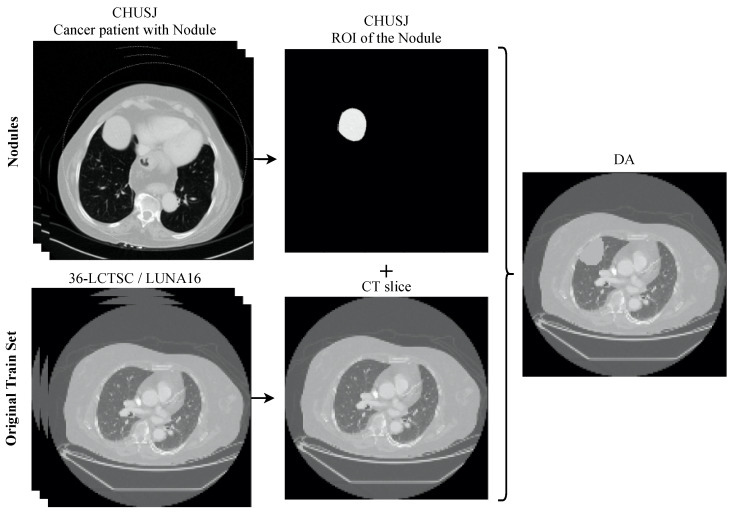
Overview of the process to generate the data augmentation. The nodules from the dataset CHUSJ were added to the original training set (36-LCTSC and LUNA16), creating new CT slices with multiple pathological imaging findings.

To perform the data augmentation of the nodules, CT scans of the CHUSJ datataset, apart from the ones used for evaluation, were analyzed in the search for lung nodules, from which fourth nodules were extracted. Once the nodules’ ROIs and their respective masks were obtained, scans from the LCTSC and the LUNA16 databases were randomly selected for the synthetic addition of nodules to the training data. An example of the produced data augmentation is shown in Figure 3, and a scheme is presented in Figure 4.

### 2.6. Experiment Design

Regarding the model hyper-parameters, in all experiments, an Adam optimizer was utilized with a learning rate of 0.0001 and a batch size of 8. The loss function used was based on the DSC given by Equation (1), in which *X* represents the ground-truth image and *Y* represents the predicted mask.

(1) $$\mathrm{DSC}=\frac{2(X\cap Y)}{X+Y}$$

As mentioned above, a first experiment had already been developed [9] in which no data correction nor data augmentation was implemented, and which served as baseline for this study. Therefore, three additional experiments were conducted that were the result of the different combinations of inclusion/exclusion of DC and/or DA. The first implemented DC but no DA, the second implemented the opposite, and lastly, the third one included both DC and DA.

Following the criteria in Sousa et al. [9], 36-LCTSC and LUNA16 were used as training data, from which 30% was used as validation data. The 24-LCTSC, the HUG-ILD, the VESSEL12, and CHUSJ databases were utilized for evaluation of the models.

### 2.7. Performance Metrics

For the evaluation of the experiments, three metrics were used: DSC, given by Equation (1), Hausdorff distance (HD), and average symmetric surface distance (ASSD) [18].The HD metric is obtained by Equation (2), in which H(A,B) is the Hausdorff distance, *A* and *B* are two distinct objects, and h(A,B) is the maximum distance of any point of A to its nearest point in B and vice-versa for h(B,A).

(2) H(A,B)=max(h(A,B),h(B,A))

The ASSD metric is obtained by Equation (3), in which ASD(A,B) is the average of distances between the points of the borders of the ground-truth *A* and the predicted mask *B*, S(A) is the set of border points belonging to *A*, S(B) is the set of border points belonging to *B*, $\sum_{s_{A}\in S\left(A\right)}\left(ds_{A},S\left(B\right)\right)$ is the sum of distances of all border points of *A* to *B*, and vice-versa for $\sum_{s_{B}\in S\left(B\right)}\left(ds_{B},S\left(A\right)\right)$, and S(A)+S(B) is the sum of all border points of *A* and *B*.

(3) $$ASD\left(A,B\right)=\frac{\sum_{s_{A}\in S\left(A\right)}\left(ds_{A},S\left(B\right)\right)+\sum_{s_{B}\in S\left(B\right)}\left(ds_{B},S\left(A\right)\right)}{|S(A)+S(B)|}$$

The DSC metric measures the level of similarity between two images, with values ranging from 0 to 1, in which 1 indicates a perfect match of the images; therefore, higher values are desired. On the contrary, the HD and ASSD are distance metrics, with values in the range 0–181.0193 mm, assuming that for an image of 128 × 128, the maximum distance between 2 distinct objects is the diagonal of that image. For these two metrics, lower values are desired.

## 3. Results and Discussion

The results obtained for each one of the four types of experiments and for each test dataset are presented in Table 3, Table 4 and Table 5 for the metrics DSC, HD, and ASSD, respectively.

**Figure 5:**
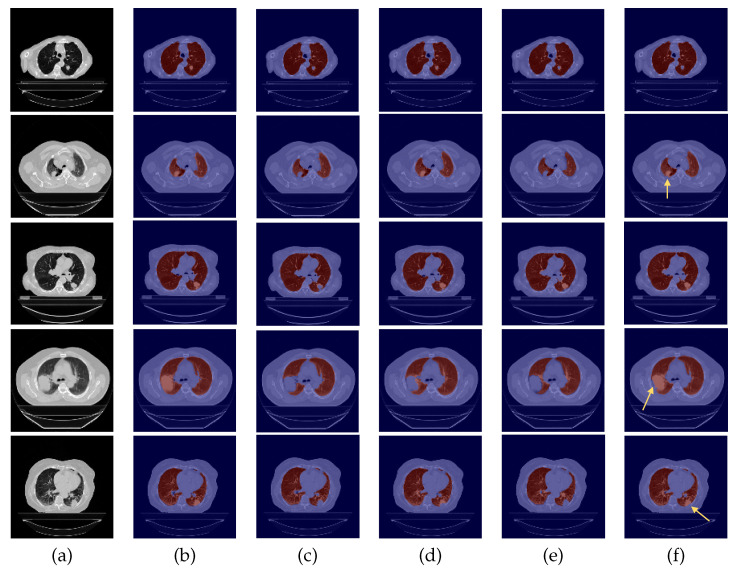
Examples of 24-LCTSC scans. From left to right: (**a**) CT original image; (**b**) ground-truth (G-T); (**c**) baseline prediction; (**d**) #2 with DC and without DA predicted mask; (**e**) #3 with DA and without DC predicted mask; (**f**) and #4 with DA and DC predicted mask.

**Figure 6:**
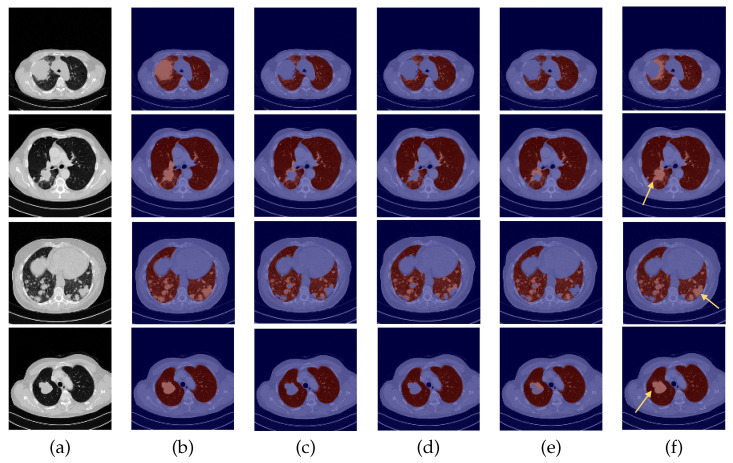
Examples of CHUSJ scans. From left to right: (**a**) CT original image; (**b**) ground-truth (G-T); (**c**) baseline prediction; (**d**) #2 with DC and without DA predicted mask; (**e**) #3 with DA and without DC predicted mask; (**f**) and #4 with DA and DC predicted mask.

**Figure 7:**
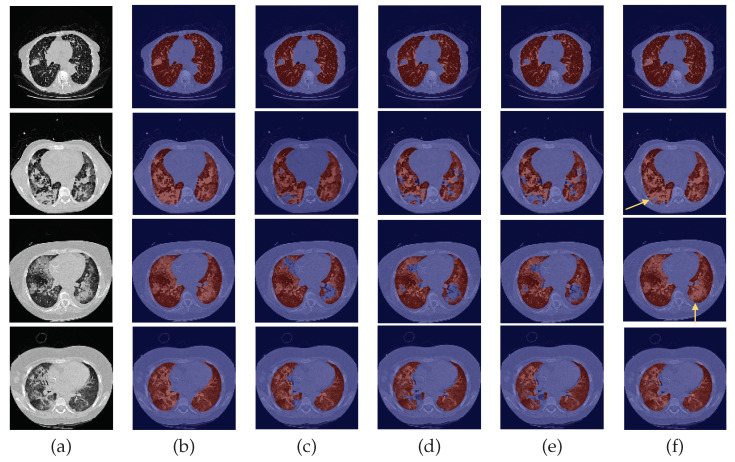
Examples of HUG-ILD scans. From left to right: (**a**) CT original image; (**b**) ground-truth (G-T); (**c**) baseline prediction; (**d**) #2 with DC and without DA predicted mask; (**e**) #3 with DA and without DC predicted mask; (**f**) and #4 with DA and DC predicted mask.

The quantitative analysis of the three metrics for the test datasets is very coherent across all four experiments, possibly due to the presence of small nodules that had little impact on the numeric results, under-segmentation of lung parenchyma and over-segmentation of other anatomical structures. Therefore, a complementary visual assessment was made to better comprehend the performance of the different models, to verify the impact of the experiments and whether there were ultimately differences between them caused by the correspondent data preparation settings employed. CT scans of the 24-LCTSC, CHUSJ, and HUG-ILD datasets are depicted in Figure 5, Figure 6 and Figure 7, respectively. Given that no differences were registered for the VESSEL12 dataset (the DA and the DC experiences did not improve or had a negative effect), no examples of these scans are displayed.

### 3.1. Experiment with DC and DA

By observing Figure 5, Figure 6 and Figure 7, one can infer that the fourth experiment, which implemented DC and DA, gave rise to the best model, despite the average numeric results. The model is more efficient to segment the lung masses in comparison to the three previous models, as it is the only one that is able to identify the whole nodule area in some cases (see third row in Figure 5 and last row in Figure 6, last column). For other images, the segmentation of this pathological element, i.e., the lung nodules, is not complete (see fourth and fifth rows in Figure 5, first, second, and third rows in Figure 6, and second row in Figure 7, last column), yet it is preferable to those produced by the remaining models. The metrics can be explained by the over-segmentation that occurred, as some masks included other surrounding anatomic structures and tissues (see last row in Figure 5, last column). Nonetheless, the overly segmented results are still preferable to masks that exclude the nodules entirely.

### 3.2. Baseline Experiment: No DC nor DA

On the contrary, the model developed in the first experiment that served as the baseline without DC nor DA, and described in detail [9], demonstrated the worst nodules segmentation results for the majority of the images (see Figure 5 and Figure 6, third columns), as a consequence of the presence of erroneous lung masks in the training dataset and the simultaneous absence of lung nodules in a substantial quantity.

### 3.3. Experiment with DC but No DA

On the second experiment, with DC and without DA, the performance is improved, as some nodules are correctly identified, as seen in the fourth column of Figure 5, row two, and the segmentation for other nodules is almost complete, as seen in the fourth column of Figure 5, rows three and five. Thus, having correct annotations is essential for the correct identification of the nodules; however, the lack of a significant presence in the training data hindered the learning of its identification.

### 3.4. Experiment with DA but No DC

With the third experiment, the one that incorporated DA but without DC, there is a decrease in the performance for the 24-LCTSC dataset, as for only one of the five displayed examples the nodule is segmented, although not completely (see Figure 5, third row, fifth column). On the other hand, for the CHUSJ dataset, the model shows the capability to identify parts of the lung nodule region (see Figure 6, second, third, and last rows, fifth column). Despite the progress registered for this last dataset and the nodule augmentation in the training data, the erroneous lung annotations, which excluded the lung masses, prevented appropriate learning. Besides that, for this model, it was verified as well that there was the misidentification of non-pulmonary structures as lung, which subsequently had a negative impact in the evaluation metrics.

## 4. Limitations

The datasets used in this work are very rich in the diversity of lung diseases, but they do not cover all the heterogeneities that can be found in clinical practice. COVID-19 is an example of a disease not covered in this training set; however, due to the similarities of this disease with other ones already used in the current dataset, the segmentation model could be robust enough to ensure a good segmentation, even in those COVID-19 cases. The data augmentation used in this work is an example of the integration of the domain of knowledge, which allows for the generation of data with more realistic features than the traditional methods that could represent limitations on their use in the healthcare field. As an example, the generation of flipped or rotated CT slices could create data that make no sense from the clinical perspective, such as hearts located in the right part of the chest, and its real world application would be questionable, even if apparently improving segmentation results. However, more complex methods for the fusion of pathological findings on the data augmentation process could be explored in future work.

## 5. Conclusions

One can conclude that the model with the superior performance was only possible due to the combination of data correction and data augmentation, as the latter can not properly work without the former. Data correction itself was demonstrated to be able to improve the performance of a model, as the inclusion of the lung masses in the training data enabled the model to learn to identify these patterns, whereas before, its exclusion indicated that these patterns were not relevant. Having correct lung annotations when performing data augmentation with the addition of nodules has proven to be crucial to the development of a successful model, since the opposite, i.e., DA in the presence of discrepancies in data, can give rise to bewildered models that will output contradictory segmentations. Once the training data were consistent, the dissemination of the nodules across it definitely served as an extra boost for its correct identification, culminating in a superior model. Nevertheless, there is still room for improvement, as the segmentation was not completely accurate for all cases.

Non-coherent annotations are a persistent issue, as annotators follow different protocols and guidelines, ultimately resulting in data that misguide the learning of the models. This work has shown the utmost importance of the homogeneity of data and, thus, hopefully, universal rules can be created that would ensure that same homogeneity in lung segmentation masks.

## Figures and Tables

**Table 1:** Datasets descriptions regarding final number of CT scans, number of slices, slice spacing, and slice thickness.

Dataset	# CTs	Number of Slices	Slice Spacing (mm)	Slice Thickness (mm)
LCTSC [13]	60	5858	1.02 ± 0.11	2.65 ± 0.38
LUNA16 [14]	176	44,098	0.69 ± 0.09	1.60 ± 0.74
HUG-ILD [15]	112	2978	0.70 ± 0.10	1.00 ± 0.00
VESSEL12 [16]	10	4279	0.74 ± 0.09	0.88 ± 0.15
CHUSJ	27	3349	0.71 ± 0.08	3.07 ± 0.38

**Table 2:** Datasets description of pathologies included and protocols utilized in the annotation process.

Dataset	Pathologies Diagnosed	Original Mask Protocol
LCTSC [13]	Multiple pathologies of the thoracic region.	Inclusion of emphysematic, inflated, fibrotic, and collapsed (this last case can be excluded in some images) lungs, and small vessels that go outside the region of the hilum. Exclusion of main bronchus and tumor masses.
LUNA16 [14]	Lung cancer.	Masks automatically generated.
HUG-ILD [15]	Interstitial lung diseases.	Not disclosed.
VESSEL12 [16]	Alveolar inflammation, diffuse interstitial lung disease, and emphysema.	Automatically generated and manually revised when needed.
CHUSJ	Lung cancer.	Exclusion of upper airways and main bronchi. Inclusion of lung nodules.

**Table 3:** Mean and standard deviation (std) results of the Dice similarity coefficient (DSC) for each dataset and for each experiment. For simplicity purposes a number is assigned to each experiment, as indicated in the first column, *#*. The DA column corresponds to the data augmentation and the DC column corresponds to the data correction. The value x in these two columns indicates experiments without DA or DC and the value √ indicates experiments with DA or DC. The values of the DSC can lie in the range 0–1.

#	DC	DA	24-LCTSC	HUG-ILD	VESSEL12	CHUSJ
			Mean ± Std	Mean ± Std	Mean ± Std	Mean ± Std
1	x	x	0.9417 ± 0.1133	0.9334 ± 0.1609	0.9778 ± 0.0726	0.9339 ± 0.1129
2	√	x	0.9458 ± 0.1029	0.9311 ± 0.1594	0.9785 ± 0.0625	0.9364 ± 0.1115
3	x	√	0.9253 ± 0.1251	0.9270 ± 0.1622	0.9809 ± 0.0569	0.9271 ± 0.1196
4	√	√	0.9412 ± 0.1088	0.9352 ± 0.1654	0.9767 ± 0.0630	0.9360 ± 0.1121

**Table 4:** Mean and standard deviation (std) results of the Hausdorff distance (HD) for each dataset and for each experiment. For simplicity purposes, a number is assigned to each experiment, as indicated in the first column, *#*. The DA column corresponds to the data augmentation and the DC column corresponds to the data correction. The value x in these two columns indicates experiments without DA or DC and the value √ indicates experiments with DA or DC. The values of the HD can lie in the range 0–181.0193 mm.

#	DC	DA	24-LCTSC	HUG-ILD	VESSEL12	CHUSJ
			Mean ± Std	Mean ± Std	Mean ± Std	Mean ± Std
1	x	x	3.1614 ± 3.7081	5.1783 ± 5.3090	1.9395 ± 3.8953	4.0943 ± 6.9651
2	√	x	4.8517 ± 6.4032	5.2617 ± 5.4048	1.9858 ± 3.9991	3.8958 ± 6.4470
3	x	√	4.3834 ± 5.5277	5.4314 ± 5.7357	1.9028 ± 3.6274	4.2868 ± 7.0384
4	√	√	4.8844 ± 6.3457	5.4441 ± 6.2199	1.9438 ± 3.7094	3.9945 ± 6.7283

**Table 5:** Mean and standard deviation (std) results of the average symmetric surface distance (ASSD) for each dataset and for each experiment. For simplicity purposes a number is assigned to each experiment, as indicated in the first column, *#*. The DA column corresponds to the data augmentation and the DC column corresponds to the data correction. The value x in these two columns indicates experiments without DA or DC and the value √ indicates experiments with DA or DC. The values of the ASSD can lie in the range 0–181.0193 mm.

#	DC	DA	24-LCTSC	HUG-ILD	VESSEL12	CHUSJ
			Mean ± Std	Mean ± Std	Mean ± Std	Mean ± Std
1	x	x	0.3816 ± 1.2082	0.4381 ± 1.4125	0.1167 ± 0.5317	0.4639 ± 1.5110
2	√	x	0.4036 ± 1.6168	0.4429 ± 1.2223	0.1121 ± 0.2732	0.4483 ± 1.6712
3	x	√	0.8286 ± 2.6559	0.5473 ± 2.1692	0.1115 ± 0.5858	0.4741 ± 1.2826
4	√	√	0.3870 ± 1.2582	0.5643 ± 2.3712	0.1167 ± 0.2921	0.4947 ± 2.3349

## Data Availability

The data were obtained from four datasets: Lung CT Segmentation Challenge 2017 [13], LUng Nodule Analysis 2016 [14], University Hospitals of Geneva-Interstitial Lung Disease [15], and VESsel SEgmentation in the Lung 2012 [16].

## References

[1] Sung H., Ferlay J., Siegel R.L., Laversanne M., Soerjomataram I., Jemal A., Bray F. (2021). Global Cancer Statistics 2020: GLOBOCAN Estimates of Incidence and Mortality Worldwide for 36 Cancers in 185 Countries. CA Cancer J. Clin..

[2] Durham A.L., Adcock I.M. (2015). The relationship between COPD and lung cancer. Lung Cancer.

[3] Silva F., Pereira T., Neves I., Morgado J., Freitas C., Malafaia M., Sousa J., Fonseca J., Negrão E., Flor de Lima B. (2022). Towards Machine Learning-Aided Lung Cancer Clinical Routines: Approaches and Open Challenges. J. Pers. Med..

[4] Firmino M., Angelo G., Morais H., Dantas M., Valentim R. (2016). Computer-aided detection (CADe) and diagnosis (CADx) system for lung cancer with likelihood of malignancy. BioMed. Eng. OnLine.

[5] Khanna A., Londhe N.D., Gupta S., Semwal A. (2020). A deep Residual U-Net convolutional neural network for automated lung segmentation in computed tomography images. Biocybern. Biomed. Eng..

[6] He K., Zhang X., Ren S., Sun J. Deep Residual Learning for Image Recognition. Proceedings of the 2016 IEEE Conference on Computer Vision and Pattern Recognition (CVPR).

[7] Ronneberger O., Fischer P., Brox T. (2015). U-net: Convolutional networks for biomedical image segmentation. Medical Image Computing and Computer-Assisted Intervention—MICCAI 2015.

[8] Tan J., Jing L., Huo Y., Li L., Akin O., Tian Y. (2021). LGAN: Lung segmentation in CT scans using generative adversarial network. Comput. Med. Imaging Graph..

[9] Sousa J., Pereira T., Silva F., Silva M., Vilares A., Cunha A., Oliveira H. (2022). Lung Segmentation in CT Images: A Residual U-Net Approach on a Cross-Cohort Dataset. Appl. Sci..

[10] Karimi D., Dou H., Warfield S.K., Gholipour A. (2020). Deep learning with noisy labels: Exploring techniques and remedies in medical image analysis. Med. Image Anal..

[11] Tajbakhsh N., Jeyaseelan L., Li Q., Chiang J., Wu Z., Ding X. (2020). Embracing Imperfect Datasets: A Review of Deep Learning Solutions for Medical Image Segmentation. Med. Image Anal..

[12] Hofmanninger J., Prayer F., Pan J., Röhrich S., Prosch H., Langs G. (2020). Automatic lung segmentation in routine imaging is primarily a data diversity problem, not a methodology problem. Eur. Radiol. Exp..

[13] Yang J., Sharp G., Veeraraghavan H., van Elmpt W., Dekker A., Lustberg T., Gooding M. (2017). Data from Lung CT Segmentation Challenge.

[14] Setio A.A.A., Traverso A., de Bel T., Berens M.S., van den Bogaard C., Cerello P., Chen H., Dou Q., Fantacci M.E., Geurts B. (2017). Validation, comparison, and combination of algorithms for automatic detection of pulmonary nodules in computed tomography images: The LUNA16 challenge. Med. Image Anal..

[15] Depeursinge A., Vargas A., Platon A., Geissbuhler A., Poletti P.A., Müller H. (2012). Building a Reference Multimedia Database for Interstitial Lung Diseases. Comput. Med. Imaging Graph..

[16] Rudyanto R.D., Kerkstra S., van Rikxoort E.M., Fetita C., Brillet P.Y., Lefevre C., Xue W., Zhu X., Liang J., Öksüz İ. (2014). Comparing algorithms for automated vessel segmentation in computed tomography scans of the lung: The VESSEL12 study. Med. Image Anal..

[17] Bryant J., Drage N., Richmond S. (2012). CT number definition. Radiat. Phys. Chem..

[18] Yeghiazaryan V., Voiculescu I. (2018). Family of boundary overlap metrics for the evaluation of medical image segmentation. J. Med. Imaging.

